# Deletion of astroglial CXCL10 delays clinical onset but does not affect progressive axon loss in a murine autoimmune multiple sclerosis model

**DOI:** 10.1186/1742-2094-11-105

**Published:** 2014-06-12

**Authors:** Emily Mills Ko, Joyce H Ma, Fuzheng Guo, Laird Miers, Eunyoung Lee, Peter Bannerman, Travis Burns, David Ko, Jiho Sohn, Athena M Soulika, David Pleasure

**Affiliations:** 1 Institute for Pediatric Regenerative Medicine, UC Davis School of Medicine and Shriners Hospital, 2425 Stockton Blvd, Sacramento, CA 95817, USA; 2 Department of Mechanical and Aerospace Engineering, UC Davis, One Shields Avenue, Davis, CA 95616, USA; 3 Current address: CJ Cheiljedang Pharma, Center for Drug Evaluation, San 522-1, Dukpyungli, Majangmyun, Icheon, Kyunggi 448130, Korea

**Keywords:** Astroglia, Axon, Conditional deletion, CXCL10, CXCR3, Experimental autoimmune encephalomyelitis, Myelin, Lymphocyte

## Abstract

Multiple sclerosis (MS) is characterized by central nervous system (CNS) inflammation, demyelination, and axonal degeneration. CXCL10 (IP-10), a chemokine for CXCR3^+^ T cells, is known to regulate T cell differentiation and migration in the periphery, but effects of CXCL10 produced endogenously in the CNS on immune cell trafficking are unknown. We created floxed *cxcl10* mice and crossed them with mice carrying an astrocyte-specific Cre transgene (mGFAPcre) to ablate astroglial CXCL10 synthesis. These mice, and littermate controls, were immunized with myelin oligodendrocyte glycoprotein peptide 35-55 (MOG peptide) to induce experimental autoimmune encephalomyelitis (EAE). In comparison to the control mice, spinal cord CXCL10 mRNA and protein were sharply diminished in the mGFAPcre/CXCL10^fl/fl^ EAE mice, confirming that astroglia are chiefly responsible for EAE-induced CNS CXCL10 synthesis. Astroglial CXCL10 deletion did not significantly alter the overall composition of CD4^+^ lymphocytes and CD11b^+^ cells in the acutely inflamed CNS, but did diminish accumulation of CD4^+^ lymphocytes in the spinal cord perivascular spaces. Furthermore, IBA1+ microglia/macrophage accumulation within the lesions was not affected by CXCL10 deletion. Clinical deficits were milder and acute demyelination was substantially reduced in the astroglial CXCL10-deleted EAE mice, but long-term axon loss was equally severe in the two groups. We concluded that astroglial CXCL10 enhances spinal cord perivascular CD4^+^ lymphocyte accumulation and acute spinal cord demyelination in MOG peptide EAE, but does not play an important role in progressive axon loss in this MS model.

## Background

Multiple sclerosis (MS) is a neuroinflammatory and neurodegenerative disease that affects more than one million people worldwide. MS and its murine model, experimental autoimmune encephalomyelitis (EAE), are mediated by activated autoreactive T lymphocytes which traffic to the central nervous system (CNS), where they are reactivated and release pro-inflammatory cytokines and chemokines, resulting in CNS recruitment and activation of innate immune cells including monocyte-derived macrophages and microglia [1-3].

CXCL10 (IP-10), a chemoattractant for many cell subsets including T lymphocytes, is upregulated in the cerebrospinal fluid and CNS lesions of MS patients [4]. Antibody-mediated systemic blockade of CXCL10 signaling has been reported to prevent recruitment of activated CD4^+^ T cells to the CNS parenchyma, and to diminish severity in an EAE passive transfer model [5]. CXCL10 has, therefore, been considered a potential therapeutic target in MS [4-6]. However, other studies have argued against this possibility. Antibody blockade of CXCL10 signaling has been reported to exacerbate EAE in an active immunization model [7], and a later study of antibody blockade of passive transfer EAE failed to confirm a diminution in disease severity [8]. Further, immunization with myelin oligodendrocyte glycoprotein peptide (MOG peptide) of mice in which CXCL10 signaling was prevented by constitutive deletion of CXCL10 or CXCR3 resulted in equal, or even greater, severity of clinical deficits than in control mice [9,10]. Immunohistological studies have shown a higher ratio of CNS parenchymal to CNS perivascular T lymphocytes in MOG peptide EAE induced in constitutive CXCR3 knockout than control mice, suggesting that CXCL10 signaling facilitates access of pathogenic T lymphocytes from CNS perivascular spaces to CNS myelin and axons [10].

The receptor for CXCL10, CXCR3, is expressed by activated T lymphocytes, natural killer (NK) cells, some dendritic cell subsets, endothelial cells, neurons [11-13], oligodendrocytes [14,15], and microglia [16,17]. Microglia in particular have been shown to migrate toward an injury site, through a CXCR3 mediated mechanism [18]. Furthermore, CXCL10-mediated microglia migration has been linked to efficient myelin debris clearance in a cuprizone-induced demyelination model [19]. There is also direct evidence that CXCL10 plays a role in effector T cell priming, with T cells from CXCL10-deficient mice exhibiting reduced interferon gamma (IFN-γ) production and decreased proliferation in response to antigen [20]. However, Lalor and Segal recently demonstrated that MOG peptide-responsive T cells generated in constitutive CXCL10- or CXCR3-knockout mice are more pathogenic than those generated in wild-type (WT) controls [21]. In addition to CXCL10, CXCR3 also recognizes CXCL9 and CXCL11. C57BL/6 mice lack functional CXCL11, while CXCL9 is expressed by microglia [22].

Although studies in constitutive CXCL10/CXCR3 knockout mice have shed light on the importance of CXCL10 in EAE, they cannot distinguish between effects of altering CXCL10 signaling on immune cell recruitment in the periphery and to, or within the CNS. To directly address this issue, we conditionally deleted CXCL10 in astroglia, the cells believed to be chiefly responsible for synthesis of CXCL10 in the EAE and MS CNS [23]. Astroglia are major players in EAE pathology and clinical disease and recent studies have shown that they control disease through the formation of a scar-like barrier [24]. However, astrocytes also express a variety of chemokines and cytokines, thus modulating the local inflammatory environment. To specifically examine the effect of astroglial CXLC10 in EAE, we compared the clinical, immunological, and neuropathological features of MOG peptide EAE in the astroglial CXCL10 knockout mice and in littermate controls in which astroglial CXCL10 synthesis remained intact. The relative accumulations of activated microglia/macrophages in areas of infiltration did not change in the presence or absence of astroglial CXCL10. Our results support prior reports showing that CXCL10 synthesized by astroglia facilitates exit of T lymphocytes from CNS perivascular spaces to the CNS parenchyma [5,10]. Surprisingly, however, we found that this CNS T lymphocyte redistribution into the CNS parenchyma was accompanied by less severe clinical deficits and demyelination in the astroglial CXCL10-deleted mice.

## Methods

### Mice

**Figure 1:**
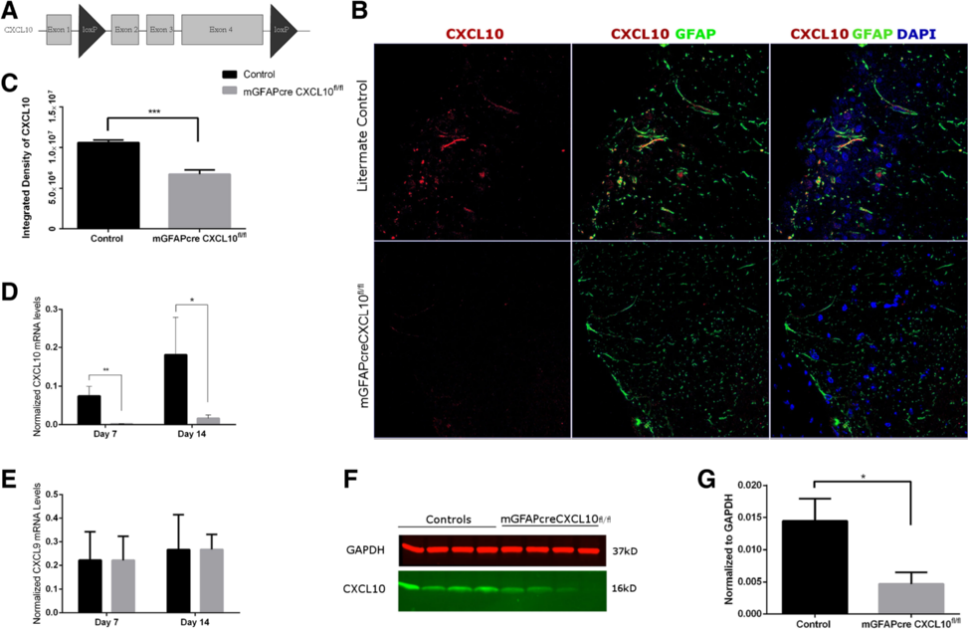
**Conditional deletion of astroglial CXCL10. (A)** Location of loxP sites in the *CXCL10* locus in CXCL10^fl/fl^ mice. **(B)** Day 17 post-MOG peptide injection (dpi) representative images of immunostaining for CXCL10-Red, GFAP-Green, and DAPI-Blue. **(C)** Quantification of images for CXCL10 integrated density at 17 dpi (n = 4–7 mice/group). **(D** and **E)** qRT/PCR for CXCL10 and CXCL9 in spinal cord of astroglial CXCL10 knockout and littermate control mice at 17 dpi (n = 6 mice/group). **(F)** Quantification of CXCL10 protein levels in the spinal cord of astroglial CXCL10 knockout and control mice at 14 dpi, by western blot analysis (n = 4 mice/group). **(G)** Data were normalized to GAPDH, *P* = 0.046. Vertical bars = SEMs.

Our lab created CXCL10^fl/fl^ mice, with loxP sites flanking *CXCL10* exons 2–3 (Figure 1A) on a C57BL/6 background. These CXCL10^fl/fl^ mice were then bred with mGFAPcre mice [25] (Jackson Laboratory) to delete astroglial CXCL10; these mice are referred to as “GFAPcreCXCL10^fl/fl^” in the manuscript. Littermate control WT mice were CXCL10^fl/fl^, but did not carry the mGFAPcre transgene. All mice were housed in a pathogen-free facility. All experimental protocols were approved by the Institutional Animal Care and Use Committee of the University of California Davis.

### MOG peptide-EAE

MOG peptide-EAE was induced in 10- to 12-week-old mice by subcutaneous flank administration of 300 μg of rodent MOG peptide (amino acids 35–55, New England Peptides) in complete Freund’s adjuvant containing 5 mg/mL killed mycobacterium tuberculosis (Difco) on day 0, with intraperitoneal administration of 200 ng of pertussis toxin on days 0 and 2. Both male and female mice were used. Previous studies in our lab have shown no significant variation between male and females in EAE onset and severity [26]. The mice were weighed and examined daily. Neurological deficits were graded on a five-point scale, as follows: 0, no detectable neurological deficits; 0.5, distal limp tail; 1.0, limp tail or waddling gait; 1.5, limp tail and waddling gait; 2.0, unilateral hind limb paresis; 2.5 bilateral partial hind limb paresis; 3.0, complete bilateral hind limb paresis; 3.5, partial hind limb paralysis; 4.0, complete hind limb paralysis; and 5, moribund or dead [3,27,28].

### RNA isolation and qRT/PCR

Mice anesthetized by intraperitoneal administration of ketamine (150 mg/kg) and xylazine (16 mg/kg) were perfused with ice-cold PBS. Spinal cords from 6 MOG peptide, and 6 normal control mice were isolated, immediately frozen on dry ice and stored at –80°C until processing. RNA was isolated from each spinal cord using RNeasy Lipid Tissue Mini Kit (QIAGEN) according to the manufacturer’s instructions and stored at –80°C. cDNA was prepared using Reaction Ready first-strand cDNA synthesis kits (SuperArray Bioscience). Real-time PCR was performed using real-time SYBR green PCR master mix (SuperArray Bioscience). Primers were designed based on PrimerBank sequences and were ordered from Integrated DNA Technologies. The mRNA levels of all genes assayed were normalized to the housekeeping gene HSP90.

### Isolation of leukocytes from mouse spleen/lymph nodes and CNS

Mice anesthetized by intraperitoneal administration of ketamine (150 mg/kg) and xylazine (16 mg/kg) were perfused with ice-cold PBS. Spleens and draining lymph nodes were harvested, combined, minced in PBS, and pushed through a 100-μm mesh. Red blood cells were lysed with ACK solution (Quality Biologicals). Brains and spinal cords were minced and digested at 37°C for 30 min in PBS containing 0.04 units of Liberase R1 (Roche) and 10 μg of DNase I (Roche) per mL. Softened fragments were pushed through a 100 μm mesh. Mononuclear cells from spleen/lymph nodes or from CNS were isolated via a discontinuous 40/70% (v/v) Percoll gradient. The cells were incubated with monesin (GolgiStop, BDBioscience) for 3 h [3,29].

### Flow cytometry

Single cell suspensions were immunostained after the incubation described above. Fc receptors were blocked with anti-CD16/32 added to the antibody staining solutions. For Th1/Th17 lymphocyte analysis, cells were stained with phycoerythrin-cyanine 7 (PE-Cy7) labeled anti-mouse CD4, and Pacific Blue (PB) labeled anti-mouse CD8, fixed, permeabilized using a Cytofix/Cytoperm Plus Kit according to the manufacturer’s protocol, and stained with allophycocyanin (APC)-labeled anti-mouse IFN-γ, PE-labeled anti-mouse IL-17 (all reagents from BD Bioscience). For T regulatory lymphocyte (Treg) analysis, cells were stained with PE-Cy7 labeled anti-mouse CD4, PB-labeled anti-mouse CD8, and APC-labeled anti-mouse CD25 fixed and permeabilized using fixation and permeabilization kits and then stained with PE-labeled anti-mouse/rat FOXP3 (all reagents from eBioscience). For macrophage subtypes and neutrophils, cells were stained brilliant violet (BV)711-labeled anti-mouse CD11b, PB-labeled Ly6G, PE-labeled Ly6C, PE-Cy7-labeled anti-mouse CD11c, PE-labeled IL-6, PE-Cy7 labeled TNFα, (BD Bioscience), APC-labeled F4/80, PE-labeled CD206, BV510-labeled CD86, PE-Cy7-labeled CD45, APC-Cy7-labeled MHCII (Biolegend), APC-labeled arginase1, biotin streptavidin-labeled Ym1 (R&D Systems), Peridinin-chlorophyll-protein complex-cyanine 5.5 (PerCP-Cy5.5)-labeled iNOS (Santa Cruz Biotechnology). Immunostained cells were analyzed using a Fortessa flow cytometer (BD Bioscience).

### Spinal cord immunohistology

Mice anesthetized by intraperitoneal administration of ketamine (150 mg/kg) and xylazine (16 mg/kg) were perfused with PBS, followed by 4% paraformaldehyde (v/v) in PBS and then post-fixed for 1 h in 4% paraformaldehyde in PBS, followed by overnight incubation in 30% sucrose and embedding in OCT. We examined lumbosacral spinal cords from healthy WT mice, CXCL10 knockout mice, and littermate control mice on days 14, 37, and 70 post-administration of MOG peptide in complete Freund’s adjuvant. OCT-embedded sections were stained with antibodies against CXCL10 (R&D, Gt, 1:250), GFAP (Gift of V. Lee, U Penn, rat 1:200), SMI 312 (Covance, Ms, 1:300), and myelin basic protein (MBP; Gift of V. Lee, U Penn, 1:3) to immunostain axons and myelin, respectively. Antibodies against laminin (Sigma, rabbit, 1:25) and against CD4 (BD, rat, 1:300) were used to delineate spinal cord perivascular spaces and CD4^+^ T cells, respectively, and slides were stained with DAPI to visualize infiltrating cells. In all cases, isotype-matched normal immunoglobulins were used as negative controls. Cross-sections through the lumbar spinal cord ventral fasciculi were analyzed for area of perivascular space and numbers of perivascular CD4^+^ T cells. Bound antibodies were detected using species- and isotype-specific fluorescently conjugated secondary antibodies and visualized by laser scanning confocal microscopy. Whole spinal cord sections were imaged using a 20x objective mounted on a Nikon laser scanning confocal microscope, stitching 20x fields of view together using Nikon NIS-Elements software. All images were acquired and processed using the same settings for all groups. Axons in the lumbar dorsal corticospinal tract and ventral fasciculus were counted with the aid of NIH ImageJ software (see counting protocol below). Myelin immunoreactivity was also measured using ImageJ.

### Semi-automated axon counting and CD4, IBA1, and MBP intensity measurements

Images of single confocal optical slices were used for axonal counting and CD4, IBA1, or MBP intensity measurements employing ImageJ software. For semi-automated axon counting we followed a previously described method [30]. Briefly, the ventral or dorsal fasciculi were selected by tracing around the area of interest, followed by splitting the colors to individual channels and selecting the appropriate (SMI 312) channel to count. We applied the drop shadows tool to highlight positive axons, and used brightness/contrast and threshold settings to adjust the image so that the smallest, dimmest countable axon in the field of view was equivalent to 3 to 4 positive pixels, and applied the watershed tool to separate any touching axons. We then used the particle analysis counter set to count from 3 to 500 pixels with circularity set to 0.2 to 1 to automatically count positive axons. To quantify MBP staining, ventral white matter tracts of individual spinal cords were outlined manually and the area lacking myelin was compared to the myelinated area to yield the percentage of demyelinated area. To quantify CD4 or IBA1 staining, ventral white matter tracts of individual spinal cords were outlined manually and the IntDen was obtained. IntDen provided a measure of intensity proportional to total volume and was calculated using area (μm) x immunoreactivity.

### Protein extraction and western blotting

Protein was extracted by adding 10 mg of spinal cord tissue to 300 μL of RIPA buffer (Santa Cruz) and sonicating at 10 amps for 10 seconds followed by 1 h incubation on ice vortexing every 10 minutes. Samples were centrifuged for 30 minutes at 4°C and supernatants were collected. Samples of 25 μg of protein in Laemmli loading buffer were analyzed under reducing conditions (BioRad Any kD gradient gel, mini-Protein precast gel), and then transferred to nitrocellulose membranes. Membranes were stained for GAPDH (Cell Signaling, Rabbit) and CXCL10 (R&D, Goat) followed by staining with fluorescently tagged secondary antibodies and imaging using a LI-COR Odyssey machine.

### Data analysis and statistics

**Figure 2:**
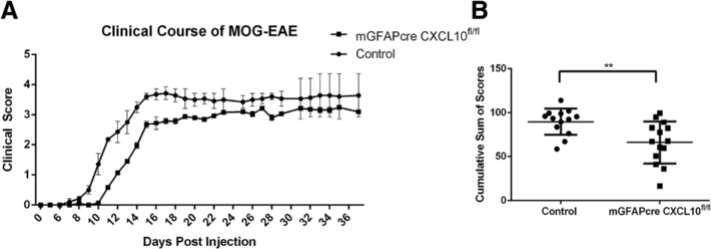
**Severity of clinical deficits in MOG peptide EAE is diminished by astroglial *CXCL10 *deletion. (A)** Average daily clinical scores and **(B)** sum of clinical scores of mGFAPcre/CXCL^fl/fl^ (CXCL10 knockout) and CXCL^fl/fl^ littermate (control) mice induced with MOG peptide EAE. Data are shown as means ± SEMs; statistical significance was determined by Wilcoxon-Mann-Whitney test, (n = 14, *P* = 0.007).

For analysis of the clinical course of disease, groups of mGFAPcre/CXCL10^fl/fl^ mice and CXCL10^fl/fl^ littermate controls received MOG peptide injections. Mice were examined daily, and clinical scores between the groups were compared using the Wilcoxon-Mann-Whitney test. qRT/PCR and flow cytometry experiment results were analyzed using Student’s *t*-tests. Immunohistochemistry images for Figures 1 and 2 were chosen as representative samples with accompanying quantification from two consecutive 14-μm lumbar spinal cord sections per mouse. SMI 312 positive axons were quantified using images of two sections per mouse. Western blot results were quantified using LI-COR software.

## Results

### Evaluation of astroglial CXCL10 deletion

To delete astroglial CXCL10, we developed a line of mice in which CXCL10^WT^ alleles were replaced by CXCL10^fl^ alleles, and crossed them with mice carrying mGFAPcre (Figure 1A).

We immunized mGFAPcre/CXCL10^fl/fl^ mice and littermate CXCL10^fl/fl^ controls with MOG peptide, and examined CXCL10 expression by immunohistochemistry 14 days later. Astroglial immunoreactive CXCL10 expression was intense in the littermate controls, but was undetectable in the mGFAPcre/CXCL10^fl/fl^ mice (Figure 1B,C). This indicates that CXCL10 was efficiently deleted in astrocytes of the mGFAPcre/CXCL10^fl/fl^ mice. Immunohistochemical analysis showed that in control mice, CXCL10 co-localized with GFAP, indicating that astrocytes were the primary source of CXCL10 expression. In astroglial CXCL10 knockout mice, while CXCL10 was undetectable in astrocytes, the overall CXCL10 immunoreactivity in whole spinal cord sections was diminished by half, and remaining positive signal co-localized primarily with infiltrating cells. qRT/PCR showed that spinal cord CXCL10 mRNA levels on days 7 and 14 post-MOG peptide immunization were substantially reduced in astroglial CXCL10 knockout mice (Figure 1D), whereas the level of mRNA encoding CXCL9, a chemokine that, like CXCL10, activates CXCR3, was not altered at these time-points in astroglial CXCL10 knockout mice (Figure 1E). Western blot analysis showed that astroglial CXCL10 knockout resulted in a 3-fold diminution in overall spinal cord levels of immunoreactive CXCL10, suggesting that, in agreement with our immunohistochemical results, CXCL10 derived from other sources such as infiltrating leukocytes and endothelial cells is intact [20] (Figures 1F,G).

### Conditional deletion of astroglial CXCL10 delays the onset of neurological deficits but does not affect the composition of immune cell infiltrates in MOG peptide EAE

In comparison to littermate controls, mGFAPcre/CXCL10^fl/fl^ mice exhibited a delayed onset and diminished severity of cumulative clinical deficits up to the end of the experiment. However, during the chronic phase of the disease, there was no significant difference in the clinical scores between the two groups, suggesting that the effect of astroglia-derived CXCL10 is likely exerted during disease initiation (Figure 2).

**Figure 3:**
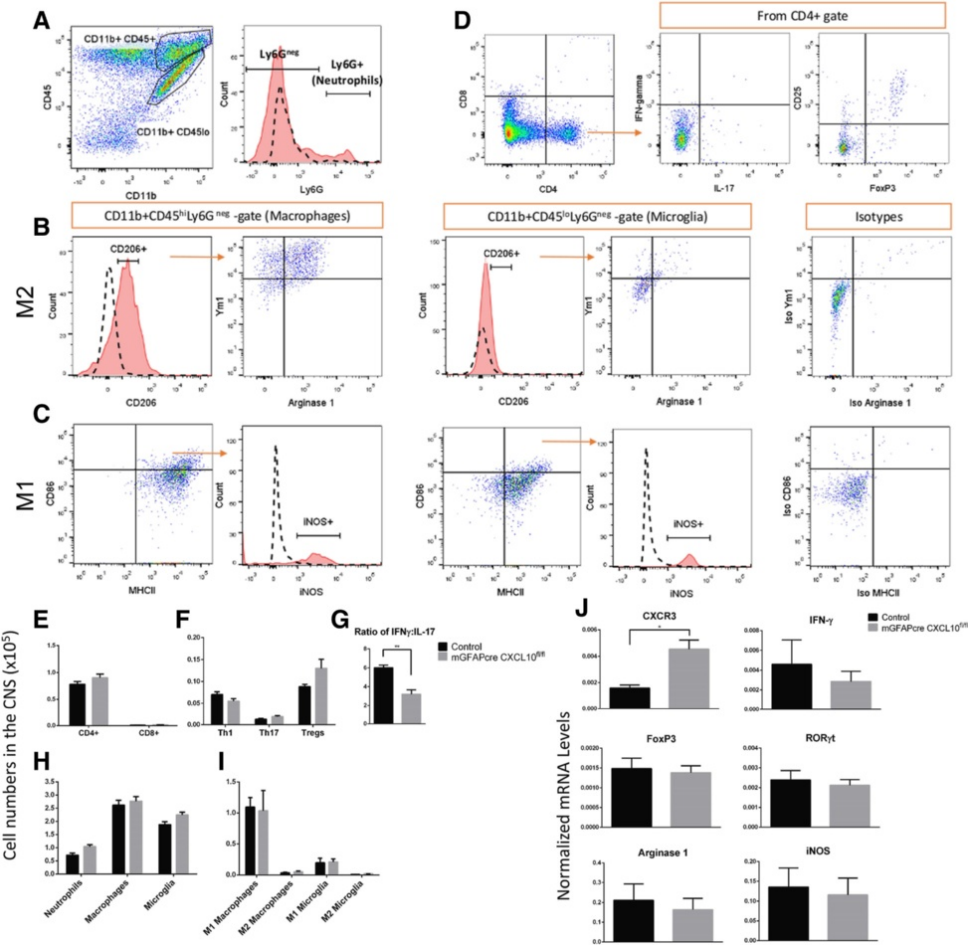
**Flow cytometry and qRT/PCR analyses of immune cell infiltrates. (A)** Flow cytometry gating strategy for macrophages (CD11b^+^CD45^hi^Ly6G^-^), microglia (CD11b^+^CD45^lo^Ly6G^-^), and neutrophils (CD11b^+^CD45^hi^Ly6G^+^). **(B)** Macrophages or microglia were further characterized as alternatively activated (M2) CD206^+^Ym1^+^Arg1^+^ or **(C)** classically activated (M1) CD86^+^MHCII^+^iNOS^+^. **(D)** Flow cytometry gating for T cell subsets: Th1 = CD4^+^IFN-γ^+^, Th17 = CD4^+^IL17^+^, Treg = CD4^+^CD25^+^FOXP3. **(E**, **F**, **G)** Flow cytometry of cells isolated from pooled brain and spinal cord showed that the only significant difference in total numbers of T cell subtypes isolated from the CNS at 21 dpi was a decrease in the ratio of IFN-γ^+^ to IL-17^+^ cells in the astroglial CXCL10 knockout mice (n = 3 mice/group, *P* = 0.0063). **(H**, **I)** No significant differences between astroglial CXCL10 knockout and control mice in total numbers of macrophages, microglia, and neutrophils **(H)** or M1 and M2 subtypes of macrophages and microglia **(I)** isolated from the CNS at 21 dpi (n = 3). **(J)** qRT/PCR of spinal cord tissue isolated at 14 dpi normalized to the housekeeping gene HSP90; CXCR3 expression was significantly upregulated in astroglial CXCL10 knockout mice compared to controls (*P* = 0.0157). IFN-γ, FOXP3, RORγt, iNOS, and arginase-1 mRNA levels were not significantly different between astroglial CXCL10 knockout and control mice at 14 dpi (n = 6 mice/group). Vertical bars = SEMs.

Flow cytometric comparisons of single cell suspensions isolated from the CNS of mGFAPcre/CXCL10^fl/fl^ and littermate control mice showed no significant differences, with the exception of a 2-fold diminution in the ratio of IFN-γ-expressing to IL-17-expressing CD4^+^ T cells in the astroglial CXCL10 knockout mice. There was no significant change in the number of total macrophages or microglia, nor in the subpopulations of CD86/MHCII/iNOS triple positive M1, or CD206/arginase-1/Ym1 triple positive M2 cells (Figure 3). Flow cytometry analysis of the spleen and lymph node tissues revealed no change in T cell, or macrophage populations between astroglial CXCL10 knockout mice and controls (data not shown).

**Figure 4:**
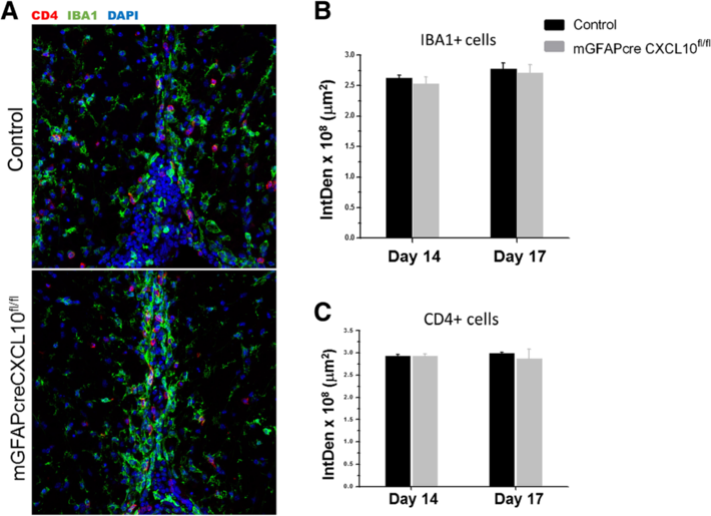
**Astroglial CXCL10 deletion does not affect microglia/macrophage accumulation within and around inflammatory lesions. (A)** Representative cross-sectional images of lumbar spinal cord ventral fasciculi of an astroglial CXCL10 knockout and a control mouse 17 dpi showing microglia/macrophage (IBA1; green) and T cell (CD4; red) accumulation in an inflammatory lesion. **(B)** Integrated Density of IBA1 and **(C)** CD4 within the lesions. There were no statistically significant changes in the IntDen measurements between the two groups.

qRT/PCR quantification of mRNAs encoding IFN-γ, the T cell transcription factors FOXP3 and RORγt, the M1 macrophage marker iNOS, and the M2 macrophage marker arginase-1 showed no differences between lumbar spinal cord of astroglial CXCL10 knockout and littermate control mice at day 14 post-MOG injection (Figure 4). Interestingly, mRNA levels of the chemokine receptor CXCR3 were higher in mGFAPcre/CXCL10^fl/fl^ mice as compared to littermate controls. The reduced CXCR3 mRNA levels in the control mice were likely due to mRNA degradation or downregulation of gene expression following internalization of CXCR3 after CXCL10 binding [31-33].Furthermore, immunohistological examination of spinal cords did not show any statistically significant differences in the number of activated microglia/macrophages between the groups in and around the infiltrated areas (Figure 2).

### CD4+ T cell localization in the inflamed spinal cord is dictated by astroglial CXL10

**Figure 5:**
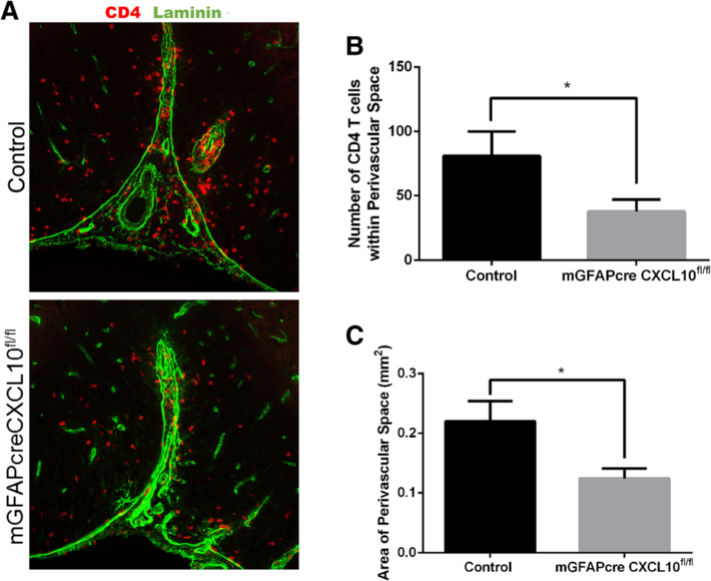
**Astroglial *CXCL10 *deletion diminishes the acute accumulation of CD4**^**+ **^**lymphocytes in spinal cord perivascular spaces. (A)** Representative cross-sectional images of lumbar spinal cord ventral fasciculi of an astroglial CXCL10 knockout and a control mouse 17 dpi, with perivascular spaces delineated by immunoreactive laminin (green) and CD4 (red). **(B)** Quantification of CD4^+^ T cells within the perivascular space surrounding the anterior spinal artery (*P* = 0.047). **(C)** Quantification of the area within laminin (n = 4–7 mice/group; *P* = 0.018). Vertical bars = SEMs.

Astroglial CXCL10 knockout altered the distribution but not the numbers of CD4^+^ lymphocytes in the spinal cord (Figures 4 and 5). Whereas in controls there was a substantial accumulation of CD4^+^ lymphocytes in the laminin-delineated spinal cord perivascular spaces, both perivascular space area and the numbers of perivascular CD4^+^ lymphocytes were substantially diminished in the astroglial CXCL10 knockout mice (Figure 5). These data are in agreement with a prior report by Müller et al. [10], who detected a similar change in the distribution of infiltrating cells using mice in which the CXCL10 receptor, CXCR3, was constitutively deleted.

### Acute spinal cord demyelination, but not chronic spinal cord axon loss, is diminished by astroglial CXCL10 knockout

**Figure 6:**
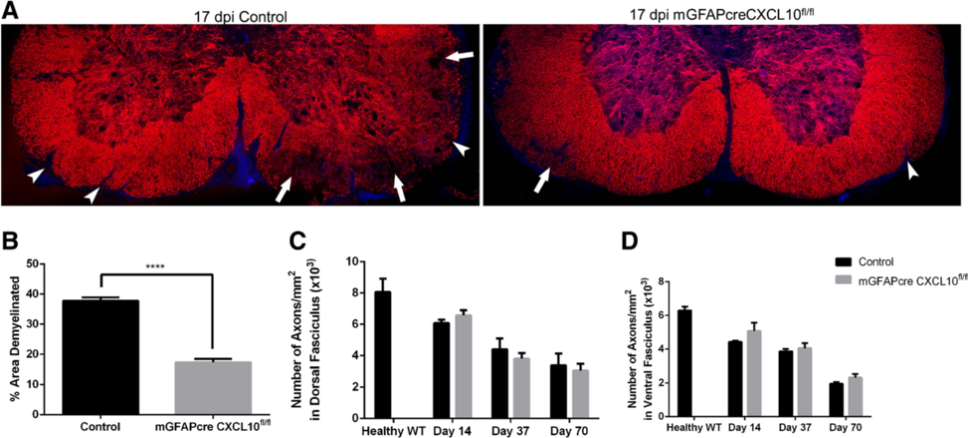
**Astroglial *CXCL10 *deletion diminishes acute spinal cord demyelination, but does not alter progressive spinal cord axon loss. (A)** Representative images of myelin basic protein (MBP, red) immunoreactivity in lumbar spinal cord cross-sections in astroglial CXCL10 knockout mice and littermate controls at 17 dpi. White arrowheads and arrows indicate small and large demyelinative lesions, respectively. **(B)** Quantification of demyelinated regions (n = 4 mice/group; *P* <0.0001). **(C)** No significant differences between astroglial CXCL10 knockout and control mice in numbers of SMI 312 positive axons in the dorsal fasciculus **(C)** and ventral fasciculus **(D)**; n = 3–7 mice/group. Vertical bars = SEMs.

Spinal cord demyelination, assessed by quantifying areas of spinal cord white matter in which immunoreactive MBP had been lost, was substantially less in day 17 post-MOG peptide mGFAPcre/CXCL10^fl/fl^ mice than in littermate controls (Figure 6A,B). There was a substantial diminution in the numbers of SMI 312+ axons in spinal cords of the mice sacrificed late after MOG peptide immunization; the extent of this loss did not differ significantly between the mGFAPcre/CXCL10^fl/fl^ and littermate control groups (Figure 6C,D).

## Discussion

To address the effect of astroglial-derived CXCL10 on the onset and progression of EAE, we engineered CXCL10^fl/fl^ mice, and crossed them with mGFAPcre mice [25] in order to conditionally delete CXCL10 in astrocytes. In this previously unavailable line of mice, CXCL10/CXCR3 signaling in the CNS is suppressed, whereas CXCL10/CXCR3 signaling in the periphery remains intact. In contradistinction to prior observations in constitutive CXCL10 knockout and CXCR3 knockout mice, the onset of clinical neurological deficits was delayed in astroglial CXCL10 knockout mice following MOG peptide immunization; however, during the chronic phase of EAE there was no statistically significant difference in the clinical severity between the groups.

Our next step was to investigate the effects of astroglial CXCL10 deletion on the number and composition of inflammatory infiltrates during the acute phase of MOG peptide EAE. Overall, CNS CD4^+^ lymphocyte accumulation during the acute phase of MOG peptide EAE was not perturbed by ablating astroglial CXCL10, possibly because there was sufficient residual CNS CXCL10 production by cells other than astroglia, and/or because, in addition to CXCR3-mediated chemoattraction, T lymphocytes can traffic to CNS by an alternative, CCL20/CCR6-mediated pathway [34]. However, we did observe two significant differences between astroglial CXCL10 knockout and control mice in CNS accumulation of CD4^+^ T cell subsets. First, there was a decrease in the ratio of Th1:Th17 lymphocytes. Second, as had previously been reported in constitutive CXCR3 knockout mice [10], astroglial CXCL10 knockout diminished the accumulation of CD4^+^ T cells in CNS perivascular spaces. Diminished CD4^+^ lymphocyte accumulation in CNS perivascular spaces did not correlate with heightened severity of EAE, as had previously been hypothesized [10]. It seems likely, instead, that increased severity of EAE in mice in which CXCL10/CXCR3 signaling has been constitutively ablated is a consequence of an alteration in systemic CD4^+^ lymphocyte priming or activation.

We detected no effects of astroglial CXCL10 knockout on acute CNS accumulations of neutrophils and macrophages, nor in the relative proportions of microglia or macrophages expressing M1 vs. M2 differentiation markers. We also confirmed that astroglial CXCL10 deletion did not have any effect on the number or composition of peripheral immune cell populations. We found a significant increase in the expression of CXCR3 mRNA in spinal cords of the astroglial CXCL10 knockout mice compared to controls. This is likely because CXCR3 is internalized and degraded following binding to any of its ligands [33,35]. Receptors may rapidly desensitize and internalize following ligand binding in order to prevent overstimulation during an immune response, with a corresponding reduction in mRNA levels as seen with type 1 angiotensin II receptor expression in smooth muscle cells [31] as well as in IL-3/CCR3 binding in eosinophils [32].

To explore the neuropathological basis for the later onset and more severe clinical deficits in control than astroglial CXCL10 knockout mice, we compared extents of spinal cord demyelination and spinal cord axon loss in the astroglial CXCL10 knockout and control mice. Acute spinal cord demyelination was less severe in the absence of astroglial CXCL10, whereas the course of spinal cord axon loss was indistinguishable between the two groups of mice. Interestingly, a previous study showed that astroglial ablation in a cuprizone model of demyelination prevents myelin loss but does not protect axons from damage [19]. The authors elegantly showed that the immunoreactive myelin in mice without astrocytes was damaged but that myelin debris was not efficiently cleared by microglia. The study further showed that microglia are likely recruited to the demyelinating site by astroglial CXCL10. To examine if that was the case, we analyzed the accumulation of IBA1+ cells (microglia and infiltrating macrophages) within the inflammatory lesion by immunohistochemistry. However, our data show that the relative intensity of IBA1+ immunoreactive cells within the lesions was not different between the groups, likely due to the fact that in EAE microglia/macrophage migration may be affected by other signals [36].

An alternative explanation would be that astrocytic CXCL10 signaling through neuronal or oligodendroglial CXCR3 may result in increased initial myelin loss without significantly affecting permanent axon loss [11-13,15]. Previous research shows that lack of CXCL10/CXCR3 signaling diminished neuronal cell death in response to NMDA-induced cytotoxicity through a microglia dependent mechanism [37]. It has also been shown that CXCL9 and CXCL10 can induce ERK1/2 activation in mouse cortical neurons, which may promote their survival, but has also been associated with neuronal cell death in models of epilepsy, ischemia, and Alzheimer’s disease [38-41].

## Conclusions

In conclusion, our data suggest that astroglial CXCL10 influences the subtypes of CD4^+^ lymphocytes that infiltrate the CNS during MOG peptide EAE, and their distribution between CNS perivascular spaces and CNS parenchyma, without significantly altering numbers or M1/M2 polarization of CNS macrophages and microglia that accumulate in the lesions. The delayed onset of neurological deficits in the astroglial CXCL10 knockout mice may, therefore, have been a consequence of delayed demyelination in the absence of astroglial CXCL10. The lack of a long-term axon-sparing effect in mice with astroglial CXCL10 ablation, as shown in the present study, argues against pharmacological modulations of CXCL10/CXCR3 signaling as a means by which to slow axon loss and disease progression in MS.

## Abbreviations

APC: Allophycocyanin; BV: Brilliant Violet; CNS: Central nervous system; Cy7: Cyanine 7; EAE: Experimental autoimmune encephalomyelitis; IFN-γ: Interferon-gamma; mGFAPcre: cre driven by mouse glial fibrillary acidic protein promoter; MBP: Myelin basic protein; MOG peptide: Myelin oligodendrocyte glycoprotein peptide 35-55; PB: Pacific Blue; PE: Phycoerythrin; WT: Wild-type.

## Competing interests

The authors declare that they have no competing interests.

## Authors’ contributions

EMK, is the first author of this manuscript, conducted most of the experiments, and wrote initial drafts of the manuscript. JHM, co-first author of this manuscript, designed the floxed CXCL10 allele and generated the conditional knockout line with the assistance of the Mouse Biology Program at UC Davis. MM, FG, LM, EL, PB, TB, DK, and JS assisted EMK in learning and performing the assays used in this study. AMS and DP are co-senior authors of this manuscript, supervised the work of EMK and the other authors, and assisted EMK in preparation of the final manuscript. All authors reviewed and approved the manuscript.

## Authors’ information

EMK and JHM are co-first authors.
